# Ions with Ions, Entities with Entities: A Proof-of-Concept Study Using the SELM‑1 Yeast Certified Reference Material for Intra- and Extracellular Se Quantification via Single-Cell ICP-Mass Spectrometry

**DOI:** 10.1021/acs.analchem.5c01588

**Published:** 2025-06-07

**Authors:** Antonio Bazo, Eduardo Bolea-Fernandez, Ana Rua-Ibarz, Maite Aramendía, Martín Resano

**Affiliations:** Department of Analytical Chemistry, Aragon Institute of Engineerinag Research (I3A), 16765 University of Zaragoza, Zaragoza 50009, Spain

## Abstract

In this work, two novel nanoparticle (NP)-based calibration strategies, external calibration and a relative method, have been explored for single-cell ICP-mass spectrometry (SC-ICP-MS) analysis. The fundamental principle of these methods is to rely on individual entities (well-characterized NPs of the target analyte) for calibration rather than on ionic standard solutions. The performance of the NP-based calibration approaches has been compared to that of the reference method (particle size with AuNP standards). In addition to the intracellular Se content (mass per individual cell), the extracellular Se (dissolved fraction) was also determined directly and simultaneously using the average background from the SC-ICP-MS time-resolved signal. The figures-of-merit of the methods developed have been evaluated by relying on the analysis of the SELM-1 cell-certified reference material, consisting of Se-enriched yeast cells, and certified for its total Se content (intracellular + extracellular Se). All methods successfully determined the Se elemental contents, but an improvement in accuracy and precision was observed for the NP-based methods compared to the reference one. Furthermore, the NP-based methods were found to be less time-consuming, more straightforward, and more user-friendly in terms of calculations. These results open new avenues for calibration in quantitative SC-ICP-MS analysis and call for a fundamental change in the methodology, where the determination of ionic contents is based on the use of ionic standard solutions for calibration, while the determination of elemental contents in discrete micro/nanoentities, such as cells, should ideally be based on calibration using standard entities, thus avoiding the need to calculate a transport efficiency coefficient.

## Graphical abstract

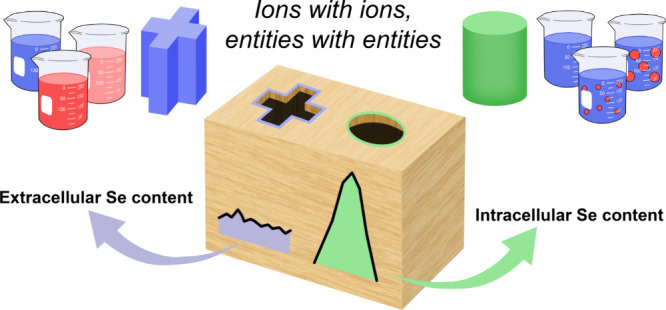

## Introduction

Inductively coupled plasma-mass spectrometry (ICP-MS) is the most powerful technique for (ultra)­trace elemental analysis in a large variety of sample types.^1,2^ Initially, the technique was developed to analyze homogeneous aqueous solutions, which was suitable for obtaining the bulk composition of a sample. However, unprecedented detection capabilities down to the attogram level (10^–18^ g) and advancements in data acquisition speeds (10 – 100 μs dwell time) have led to a shift in focus. Today, ICP-MS is also ideally suited for the analysis of discrete entities, such as engineered nanoparticles, microplastics, and single cells.^3^ Compared to the former bulk approach, ICP-MS operated in single-event mode relies on the introduction of a highly dilute heterogeneous aqueous suspension containing micro/nanoentities, giving rise to a time-resolved signal with individual events whose integrated intensities are proportional to the mass of the target analyte within the micro/nanostructures.^4–6^ This methodology was first deployed for the analysis of colloids and nanoparticles (NPs), but over the last years, it has been realized that this approach could also be of more general application.^7–11^ Among the different available options, the analysis of individual cells via single-cell ICP-MS is particularly noteworthy given the possibility of obtaining the mass distribution of the analytes present in the cell (either endogenous or imported by cellular exposure) rather than their average content,^12^ which is especially critical in fields such as medicine and biochemistry.^13,14^ Another advantage of the single-event mode that is often underexplored is the possibility of obtaining direct quantitative information on the fraction of analyte present in the form of micro/nanoentities (signal events) and that dissolved in the media (average background signal). In the case of cells, this is particularly relevant, as it allows obtaining both the intra- and extracellular content, providing a comprehensive picture of cell biology and its microenvironment.

The characterization of NPs and the analysis of single cells by single event-ICP-MS (referred to as SP-ICP-MS and SC-ICP-MS, respectively) share many similarities, but the level of understanding and the number of applications developed for both techniques are very different, with the former being significantly more mature. However, the rapid and sometimes parallel growth of both approaches has given rise to a paradigmatic situation, in which the same quantitative strategies are often applied in both cases without fully considering the significant differences existing between NPs and cells. Cells are not only more fragile and much larger than NPs, but they also exhibit a much more complex chemical composition with vastly lower analyte contents.^15^ Despite these differences, the same calibration approaches originally developed for SP-ICP-MS are being used for SC-ICP-MS, without carrying out a critical evaluation of the convenience of deploying such approaches. This paper seeks to correct this situation, as well as to provide calibration approaches specifically designed for SC-ICP-MS analysis.

Among the different calibration approaches that have been proposed in the literature for the characterization of NPs by SP-ICP-MS,^16–18^ SC-ICP-MS analysis generally relies on the so-called particle size method to correlate the integrated intensity of the events with the mass of the analyte contained in the corresponding cells. This method is based on the use of ionic standard solutions for calibration and requires the determination of the transport efficiency (TE) with a reference NP for the calculations. In SP-ICP-MS, it is often assumed that ionic standard solutions and NPs show very similar behavior in the ICP. However, recent works have reported more imprecision and biases for ionic-based calibration approaches as compared to those directly relying on discrete entities (e.g., NP standards) for calibration,^18–20^ which, among other reasons, could point to a not-so-similar behavior of ions and entities in the ICP. It seems obvious that the behavior of ions and cells can differ massively, and thus, exploring alternative calibration methods for SC-ICP-MS analysis based on the use of entities seems rather interesting. To the best of the authors’ knowledge, such methods have not been implemented for the determination of ionic analytes in cells, but only to determine the number of NPs introduced in the cell by comparing the sensitivity of cell events with those of the NPs.^21–24^ Moreover, the SC-ICP-MS calibration strategies adopted from the SP-ICP-MS technique are prone to result in bias, are generally time-consuming, and are still far from routine application.

However, given the difficulties associated with obtaining accurate SC-ICP-MS results, proving that the improvements already observed for the characterization of NPs by using TE-independent calibration methods will directly translate to SC-ICP-MS results is not an easy task. As for any other technique, developing novel quantitative calibration strategies strongly depends on the possibility of validating such methods. Validation of SC-ICP-MS results is often based on digesting a well-known number of cells that are subsequently analyzed for the bulk content of the analyte of interest. This approach, however, suffers from various limitations, such as the large uncertainty associated with the determination of cell number concentrations, the potential effect of contamination that results in overestimations, and the impossibility to distinguish between the intra- and extracellular content. Moreover, even when successful, bulk analysis only provides the average concentration without considering the cell-to-cell heterogeneity within a cell population. Fortunately, the growing interest in the field of cellular analysis has encouraged the development of cell-certified reference materials (CRM), such as SELM-1 (National Research Council Canada, NRC), which consists of selenium-enriched yeast cells certified for total Se content (intracellular + extracellular Se). Therefore, this CRM is a perfect model to (*i*) try and validate for the first time SC-ICP-MS results obtained with TE-independent calibration methods based on the use of NP standards, taking Se as a model element, (*ii*) to compare these results with those achieved by the more conventional approach (based on the determination of the TE with the particle size method and AuNP standards), and (*iii*) to evaluate the direct and simultaneous determination of both the intra- and extracellular Se content. This will also help demonstrate the applicability and possibilities of such a methodology.^25^

In this work, both intra- and extracellular Se contents in yeast cells are accurately, directly, and simultaneously determined for the first time from the time-resolved SC-ICP-MS signal. For the quantitative determination of the extracellular content, the average intensity of the background of the cell analysis is directly interpolated in an ionic calibration curve (ions with ions). On the other hand, for the intracellular Se content, two novel TE-independent approaches based on the use of NP standards of the target analyte for calibration (external calibration and a relative method) are proposed. These approaches directly compare Se events coming from NP standards of well-characterized mass with those of the yeast cells (entities with entities). It is important to state that such entities might exhibit different behavior in terms of TE due to their differences in size and properties, but as long as both are completely ionized within the plasma, their intensities should be comparable. In other words, the assumption is not that the TE of cells and NPs is similar. The assumption is that, whenever these discrete entities reach the plasma, they provide comparable sensitivity.

The performance of these methods during five different working sessions is evaluated by comparing the results obtained in each case with those of the reference method (particle size). The total Se content (intracellular + extracellular Se) determined with each approach is finally validated by comparison with the certified Se concentration of the SELM-1 reference material.

## Materials and Methods

### Reagents, Standards and Samples

Ultrapure water (resistivity >18.2 MΩ cm) was obtained from a Milli-Q water purification system (Millipore, France). Reagents of analytical purity grade were used throughout this work. Single-element standard solutions of Au and Se (1 g L^–1^, Merck, Germany) were respectively diluted in 0.6 M HCl and 0.14 M HNO_3_ solutions prepared by appropriate dilution from 12 M HCl and 14 M HNO_3_ stock standard solutions (Suprapur, Merck) with Milli-Q water.

Appropriate dilutions of the original NP suspensions – 60 nm AuNPs (60 ± 3.5 nm; HiQ-Nano, Italy) and 150 nm (average: 150 nm; range: 140 nm – 160 nm) and 250 nm (average: 236 nm; range: 230 – 270 nm) SeNPs (Merck) – were prepared in Milli-Q water and used for calibration. SELM-1, a selenium-enriched yeast CRM from NRC with information on total selenium content, was used for method validation. For bulk analysis, 14 M HNO_3_ (Suprapur, Merck) and 9.8 M H_2_O_2_ (VWR, Belgium) were used for sample digestion.

### Instrumentation

All measurements were carried out using a NexION 5000 (PerkinElmer, USA) ICP-MS/MS instrument. The triple cone interface with OmniRing^TM^ was operated in extraction mode to achieve the maximum sensitivity. This instrument is equipped with a quadrupole ion deflector (QID, Q0) that selectively focuses the ion beam over a 90-degree angle, before introduction into the mass spectrometer, which consists of two additional quadrupole units (Q1 and Q3) and a quadrupole collision/reaction cell (CRC; Q2) located in between. Since the ^82^Se isotope (relative abundance of 8.7%), which is less affected by the occurrence of spectral interferences than the most abundant ^80^Se isotope, was monitored in this work, the instrument was operated in single-quadrupole or “Q3 Only” mode. The limit of detection (LoD), calculated as 3 times the standard deviation of the blanks divided by the slope of the calibration curve, was found to be 0.26 fg.

A Harvard Pico Plus 11 Elite low-flow syringe pump (Harvard Apparatus, USA) equipped with 1 mL (0.01 mL – 1 mL) sterile syringes (Henke Sass Wolf, Germany) enabled sample introduction at a flow rate of 20 μL min^–1^. The high-efficiency sample introduction system (Single-Cell Sample Introduction Kit, PerkinElmer) comprised a CytoNeb with PFA gas line nebulizer fitted onto the Asperon spray chamber. The Asperon was developed specifically to increase the TE of cells to the plasma by incorporating new flow patterns. This includes a dual makeup gas inlet that creates a tangential flow to the spray chamber walls to prevent cells from colliding and an inner tube with microchannels to prevent liquid deposition in the flow path.

To ensure a gentle introduction of yeast cells without sacrificing instrument sensitivity, the nebulizer and makeup gas flow rates were optimized and set at 0.3 and 0.9 L min^–1^, respectively. The single-cell module of the Syngistix software (version 3.5) was used for SC-ICP-MS analysis and for a first visualization of the results.

Daily checks were carried out to ensure optimum instrument performance. This includes optimization of the torch position, QID, and gas settings, so that the instrument sensitivity was maximized while keeping the Ce^2+^(70)/Ce^+^(140) and CeO^+^(156)/Ce^+^(140) ratios equal or under 0.03 and 0.025, respectively. Instrument settings and data acquisition parameters are listed in Table S1 of the Supporting Information. Information on cell numbers was obtained using a phase contrast optical microscope (Nikon Eclipse E400, Nikon Inc., Japan) and a Neubauer Thoma cell counting chamber (Avantor, USA).

### Sample Preparation

To evaluate the performance of the different methods, the SELM-1 CRM (Se-enriched yeast cells) was characterized within five different working sessions. For each SC-ICP-MS working session, fresh sample and standard dilutions were prepared from the stock material. In this context, NP standards were diluted until a PNC of approximately 3 × 10^5^ NPs mL^–1^ was achieved, so that the probability of facing double events was kept below 0.1%. For the cells, and following the protocol described in the certificate, approximately 20 mg of the SELM-1 CRM was suspended in 10 mL of Milli-Q water with the aid of a vortex (>1 min). The cell suspension was further diluted (100-fold) in Milli-Q water to reach a cell density of approximately 3 × 10^5^ cells mL^–1^, similar to the NPs concentration. While the TE in number (i.e., the ratio between the number of registered events versus the number of entities introduced into the system) is lower for cells than for NPs (average TEs of 21% and 37%, respectively), the same target concentration was used to further minimize the occurrence of double events. As will be discussed below, keeping the probability of double events low is paramount for SC-ICP-MS, as the distribution model to which single cell intensities adjust is not as clear as it typically is for monodisperse commercial NPs. As a result, the average intensity of all the cell events needs to be selected as the analytical signal, and this parameter is much more affected by the presence of double events than the most frequent size, usually chosen instead for NPs.

For bulk analysis, the SELM-1 CRM was acid-digested to determine the total Se content. A fraction of 0.2 g of homogenized sample was accurately weighed in a Savillex PFA beaker, and 3 mL of HNO_3_ and 1 mL of H_2_O_2_ were added. The samples were digested on a hot plate at 110 °C overnight. Subsequently, the digests were evaporated at 70 °C until almost dry, and the residues were redissolved in 10 mL 0.14 M HNO_3_. The resulting solutions were further diluted (2000-fold) prior to solution-based bulk ICP-MS analysis.

### Data Processing

Each data set recorded during a working session was exported from the Syngystix single-cell module for data processing. Ionic data sets were directly processed to calculate their average intensity and standard deviation with the OriginPro software (version 2021b, 9.85), which was also used for plots, fittings, interpolations, and statistics throughout the work. The integration of single events was performed with the Hyper Dimensional Image Processing (HDIP v1.8.4) software. After appropriate integration, the signal intensities for NPs were fitted to Gaussian distributions, and their central value was selected as the analytical signal. However, the average intensity was used for cells, as the distribution does not necessarily follow a Gaussian shape.

## Results and Discussion

The SELM-1 Se-enriched yeast reference material is certified for its total Se content,^26^ which encompasses both the intra- and extracellular Se. In this work, a suspension of yeast cells was analyzed via SC-ICP-MS to obtain a data set enabling the determination of both elemental contents, as schematically represented in Figure 1.

**Figure 1:**
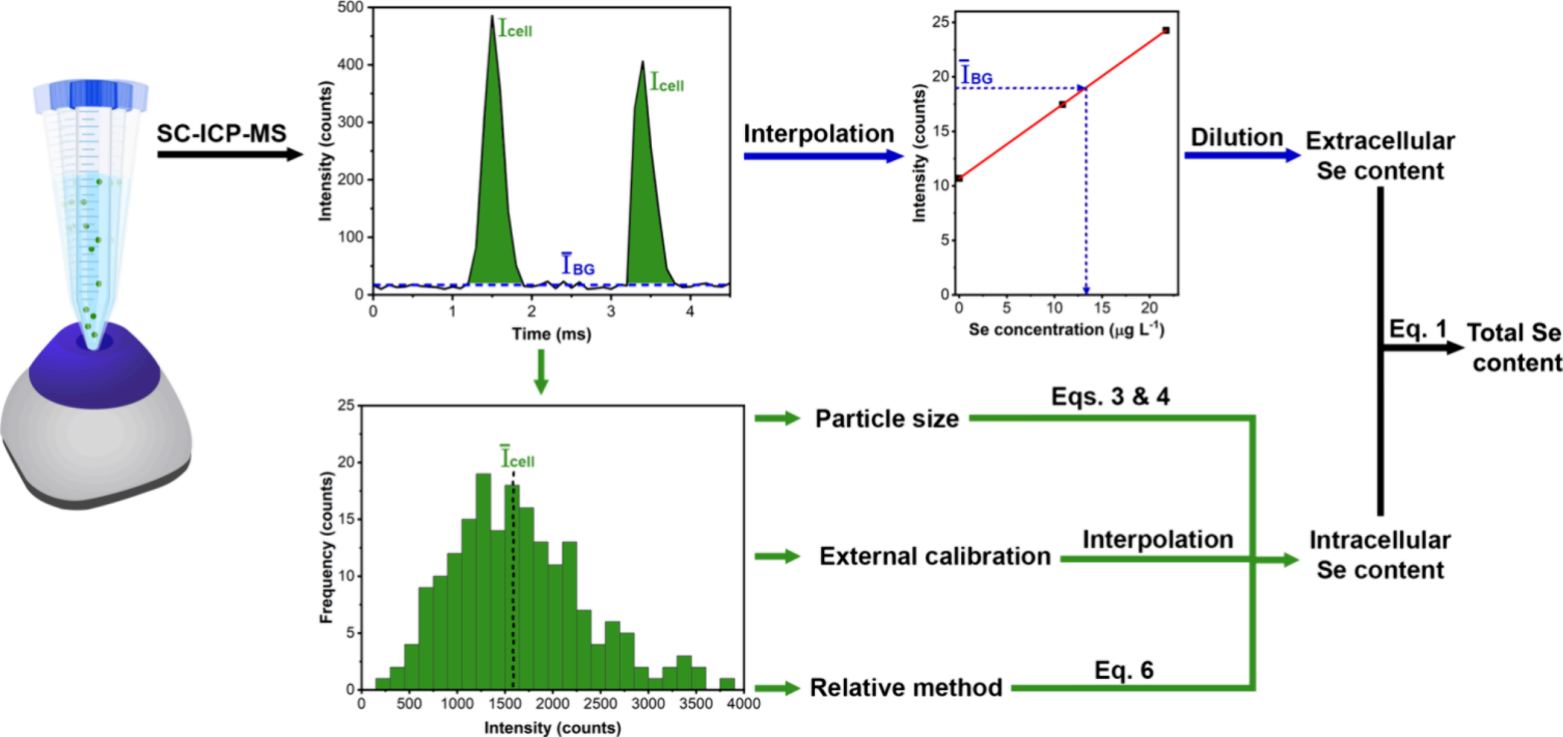
Schematic representation of the different steps performed in every working session to determine both the intra- and extracellular Se content from the data set obtained from the analysis of the SELM-1 cell suspension via SC-ICP-MS.

The intracellular content of each cell was proportional to the integrated intensity of its corresponding event, and appropriate calibration strategies were developed to calculate the average Se mass per cell (m_cell_). The extracellular Se content (m_extra_) was determined directly by interpolating the average background signal on a calibration curve constructed with ionic Se standards and applying the corresponding dilution factors.

Once both contributions to the total Se content were determined, the total Se mass per gram of sample (m_Se_) was calculated in accordance with eq 1, where N_cell_ is the number concentration of yeast cells per gram of sample (1.8 × 10^10^ ± 0.3 × 10^10^ cells g^–1^; n = 3, uncertainty expressed as standard deviation). The results could be evaluated in terms of recovery (R%) when compared to the certified Se concentration (m_CRM_), according to eq 2.

$$m_{\mathrm{Se}}=m_{\mathrm{extra}}+m_{\mathrm{cell}}N_{\mathrm{cell}}$$ 1

$$R\%=\frac{m_{\mathrm{Se}}}{m_{\mathrm{CRM}}}100$$ 2

### Intracellular Determination: Standard Method

The most common strategy for calibrating SC-ICP-MS signals consists of calculating the TE with the particle size method according to eq 3 and correlating the intensity of the cell events (I_cell_) with their corresponding mass (m_cell_) according to eq 4:

$$\mathrm{TE}=\frac{M_{\mathrm{ion}}S_{\mathrm{ion}}m_{\mathrm{NP}}60}{M_{\mathrm{NP}}I_{c,\mathrm{NP}}Q_{\mathrm{neb}}t_{\mathrm{dwell}}}$$ 3

$$m_{\mathrm{cell}}=\frac{I_{\mathrm{cell}}t_{\mathrm{dwell}}Q_{\mathrm{neb}}\mathrm{TE}}{S_{\mathrm{an}}60}$$ 4

where (m_NP_) is the NP mass, I_c, NP_ is the central value of the intensity distribution in counts, M_NP_ and M_ion_ are, respectively, the molar mass of both the standard NP material and the element monitored, S_ion_ is the sensitivity of that element in mL fg^–1^ counts^–1^, Q_neb_ is the sample uptake rate in mL min^–1^, t_dwell_ is the dwell time in seconds, and S_an_ is the analyte sensitivity.

This method has traditionally been deployed using AuNPs as reference NP standards, given the great accessibility to well-characterized AuNPs, even though Au is not always the target analyte within the cells and may thus have a very different sensitivity. However, since the sensitivity of both the analyte (S_an_) and the element composing the reference NP (S_ion_) are included in the equations applied for this method (eq 3 and 4), such differences are corrected with no further action required.^27^ This can be clearly seen in eq 5, where after substituting the TE in eq 4 with eq 3 (and assuming the same dwell time and sample flow for the measurement of both NPs and cells), a much simpler equation is obtained, introducing the correction as the quotient between both sensitivities.

$$m_{\mathrm{cell}}=\frac{I_{\mathrm{cell}}M_{\mathrm{ion}}m_{\mathrm{NP}}}{M_{\mathrm{NP}}I_{c,\mathrm{NP}}}\frac{S_{\mathrm{ion}}}{S_{\mathrm{an}}}$$ 5

By applying these equations, and using 60 nm AuNPs as the reference standard, an average TE of 42.8 ± 5.5% was obtained for the five different working sessions, leading to an average intracellular Se content of 68.9 ± 7.3 fg cell^–1^. The results (Se mass per cell) are shown in Figure 2; this figure also provides an overview of the results obtained using the two NP-based calibration strategies, as will be discussed in the following section. As to the results using the particle size method, large differences were found between working sessions, with average intracellular Se contents ranging from 60.8 fg cell^–1^ to 79.3 fg cell^–1^. These differences in the results between measurement sessions can potentially be attributed to inaccuracies in the calculation of the TE, as it should be noted that the particle size is a TE-dependent method, and these strategies have been found to be more prone to imprecision and bias than TE-independent methods.^18^ Self-evidently, other NP types different than AuNPs can be relied on for TE calculation, although a similar performance is to be expected in these situations, unless the selected reference NPs are made of the same element being determined in the cells. However, a particle size method that would meet these conditions will not really depend on ionic calibration standards and should, therefore, be better referred to as external calibration, as discussed in the next section.

**Figure 2:**
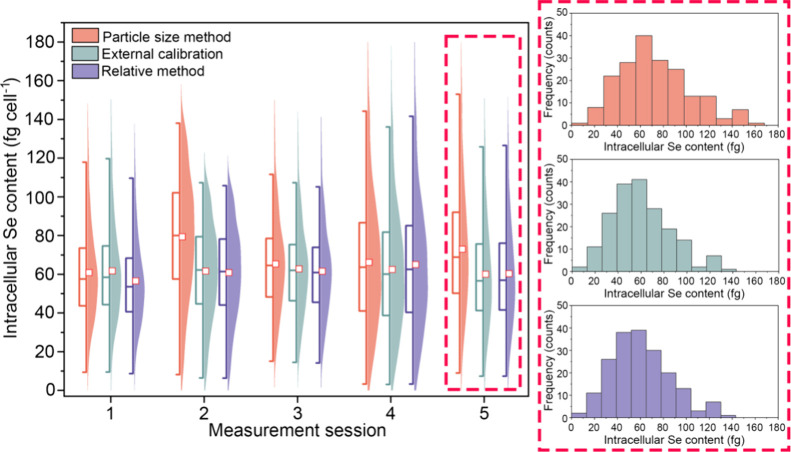
Box and half-violin plot of the results obtained for the determination of the intracellular Se content using the particle size method, external calibration, and the relative method, as calibration strategies during five measurement sessions. The blow-up shows an example of the mass distributions for Se-enriched yeast cells using the three different calibration approaches.

### Intracellular Determination: Calibration Methods Based on NPs of the Target Analyte

Two alternative NP-based calibration strategies (TE-independent) have been evaluated in this work for the quantitative determination of the intracellular Se content in yeast cells via SC-ICP-MS: (*i*) external calibration and (*ii*) a relative method. These strategies have already shown superior performance in terms of accuracy and precision compared to the particle size method for sizing NPs in a previous work.^18^

As stated above, the use of NP standards of the same target element allows for the implementation of an external calibration strategy for quantitative single-event ICP-MS analysis. These strategies are not commonly applied for the analysis of discrete entities, most likely because of the traditional shortage of suitable NP standards. However, due to the growth in the nanoparticle market, the catalogue of commercially available inorganic NPs includes an increasing variety of standards of different elements (*e.g.,* Ag, Au, B, Ca, Cu, Fe, Pt, Si, Zn, ···), which in many cases provides for the acquisition of NPs of the same analyte to be determined within cells. Moreover, the synthesis of many types of inorganic NPs can be accessible to analytical chemists as well (which opens up new ways for, *e.g.,* carrying out tracer experiments).^28^ However, the application of these approaches requires these NPs to be of well-characterized density, chemical composition, and size, as well as monodisperse enough to clearly distinguish a maximum of intensity in the registered histograms.

Taking this into account, it seems timely to assess the potential of this strategy and the possibility of changing toward a new trend of entity-based calibration strategies. The external calibration approach was based on the construction of a calibration curve intensity versus the mass of the target analyte using SeNP standards of 150 nm and 250 nm (nominal sizes; see Materials and Methods for further information) rather than ionic standard solutions. Self-evidently, the NPs used as calibration standards need to have well-defined chemical composition, density, size, and shape, to minimize the calibration uncertainty. To construct the calibration curve, a blank solution and the two SeNP standards available were measured, and the central value of their intensity distributions was plotted versus the calculated SeNP mass, leading to the calibration curve parameters collected in Table S2 of the Supporting Information (R^2^ > 0.9998). The intensity of the yeast cell events was just interpolated in this curve to derive the mass of analyte present on each detected cell (intracellular Se content). An average intracellular Se content of 61.7 ± 1.1 fg cell^–1^ was obtained for the five working sessions.

As can be seen in Figure 2, the repeatability of the results using external calibration was significantly enhanced as compared to the particle size method, with average intracellular Se contents ranging from 59.9 fg cell^–1^ to 62.7 fg cell^–1^. This improvement can be attributed to the lower uncertainty associated with the use of reference NPs for calibration, avoiding biases in the measurement of ionic standard solutions and their corresponding measurement uncertainties (TE-independent). Furthermore, the use of multiple NP standards for calibration enhances result accuracy and reliability compared to single-point calibrations, which may lead to higher uncertainty and potential bias. In this context, the repeatability of the results slightly worsened when calibration was performed using a single NP with a significantly different analyte mass (average mass per cell of 63.5 ± 2.8 fg and 61.8 ± 1.1 fg for calibration using 150 nm or 250 nm SeNPs, respectively). These differences, depending on the size of the NP standard, have already been explained in a previous work, which demonstrated that the relative error decreases when calibration is performed using NPs with a mass content closer to that of the target entity.^18^ In any case, the greater availability of NP standards of different sizes demands for a multipoint external calibration, allowing one to statistically assess potential outliers within the calibration curve affecting the linearity, such as those resulting from NPs of masses different than expected or those that may potentially not be completely digested in the ICP, enabling a more in-depth study for each specific application.

The relative method adapts the approach of Moreira-Álvarez et al.^17^ for NP sizing, extending its application to the analysis of single cells. The original approach relied on the monitoring of solutions of an ionic standard and a reference NP at different conditions. By plotting the relative drop in signal intensity of the ionic standards versus the shift of the central values of the distribution of either a reference NP or an NP sample, two curves were constructed, and their slopes could be used to obtain the mass of the NP sample.

In this work, this method has been adapted so that (*i*) there is no need to measure ionic standard solutions (NP-based) and (*ii*) it provides the mass of the target analyte within individual cells. In this case, 250 nm SeNPs and Se-enriched yeast cells (SELM-1) were monitored under different sensitivity conditions by stepwise lowering the voltage of the quadrupole ion deflector (QID) from −12 to −18 V (see Figure 3). For each measurement condition, the central intensity of the NP distributions and the average intensity of the cell distributions were evaluated to plot the shift of the central values of the Gaussian distributions obtained for 250 nm SeNPs (I_NP1_ – I_NPi_) and the average intensity of yeast cell distributions (I_c1_ – I_ci_), always using the optimum QID as reference. Then, the slope of the adjusted linear fitting (s_rel_) can be used to obtain the analyte mass per individual cell (m_cell_), in accordance with eq 6, where m_NP_ is the well-known mass of the 250 nm SeNP standard.:

$$m_{\mathrm{cell}}=\frac{m_{\mathrm{NP}}}{s_{\mathrm{rel}}}$$ 6

**Figure 3:**
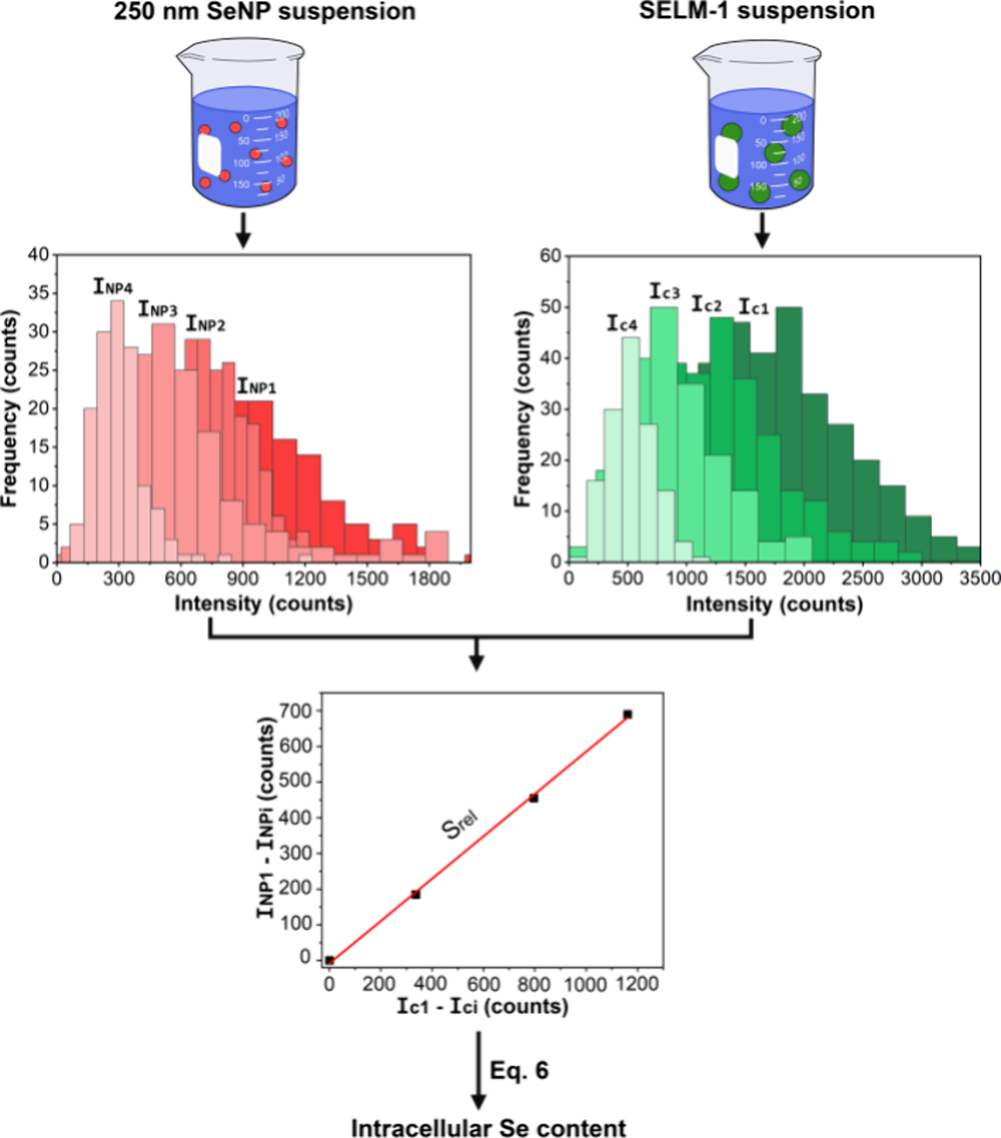
Representation of the relative method adapted from Moreira-Álvarez et al.^17^ for single-cell analysis. Subindexes represent the different sensitivity conditions (1 being the optimum).

An average intracellular Se content of 60.7 ± 3.0 fg cell^–1^ was obtained for the five working sessions. The repeatability of the results using the relative method was found to be in between that of the particle size and the external calibration approaches, with average intracellular Se contents ranging from 56.4 fg cell^–1^ to 64.9 fg cell^–1^ (see Figure 2). This intermediate performance can directly be attributed to the pros and cons of the relative method. On the one hand, this is a TE-independent method that does not depend on the measurement of ionic standards with their associated measurement uncertainties, which explains the better performance compared to the particle size method. On the other hand, external calibration still surpasses the performance of the relative approach, as it relies on the monitoring of multiple NP standards for calibration, while the relative method is based on a single NP standard. The relative method can thus be seen as a multisignal calibration strategy,^29,30^ in which the advantage is based on the monitoring of such an NP standard under multiple instrumental conditions rather than on a single measurement, which improves the robustness and minimizes instrumental uncertainty. However, the requirement of monitoring both the reference NP and the cells under progressively less sensitive conditions can also affect the accuracy due to limit of detection constraints, especially for the latter points of the curve.

Overall, the different methods provided similar results for the determination of the intracellular Se content, but the NP-based methods stand out for greater performance. As discussed below, the latter also demonstrated greater accuracy when compared with the certified total Se content after incorporating the extracellular Se. However, it should not be forgotten that the commercial accessibility, information available (composition, density, size, shape), and stability of AuNPs can still make the reference method based on particle size an attractive option.

### Extracellular and Total Se Determination

In addition to the intracellular content, SC-ICP-MS can also offer information about the extracellular one, thus providing a more comprehensive picture. In a previous work focusing on the analysis of the SELM-1 CRM, an approach based on successive steps of washing and centrifugation was developed for the determination of the extracellular content.^25^ After yeast cells’ isolation, the dissolved Se in the washing solution (extracellular fraction) was determined separately, yielding accurate results. However, a direct and simultaneous strategy for determining both the intra- and extracellular content has been evaluated in this work by monitoring not only the individual events (intracellular) but also the average signal intensity of the background, which can be correlated with the dissolved Se concentration by interpolating in an ionic calibration curve, and then to the extracellular Se content per gram of sample by applying the corresponding dilution factors.^31^ To assess the feasibility of this strategy, the extracellular contents obtained via SC-ICP-MS were compared to those of the former approach based on centrifugation. Four different SELM-1 suspensions were prepared by diluting approximately 30 mg of the sample after respectively 0, 1, 2, or 3 washing steps by centrifuging for 5 min at 600g with 5 mL of Milli-Q water, so that the supernatant (washed cells) was separated from the washing solution containing the extracellular Se. Then, both solutions were analyzed individually, leading to the results collected in Figure 4. No significant differences in the extracellular Se contents were observed between the washed (indirect analysis of the washing solutions) and unwashed samples (monitoring of the SC-ICP-MS average background signal), regardless of the number of washing steps. These results suggest that the direct approach is suitable for the determination of the extracellular Se content in the SELM-1 CRM, as it provides accurate results and minimizes sample preparation. Therefore, the direct approach was selected for further validation of the total Se content in the SELM-1 CRM.

**Figure 4:**
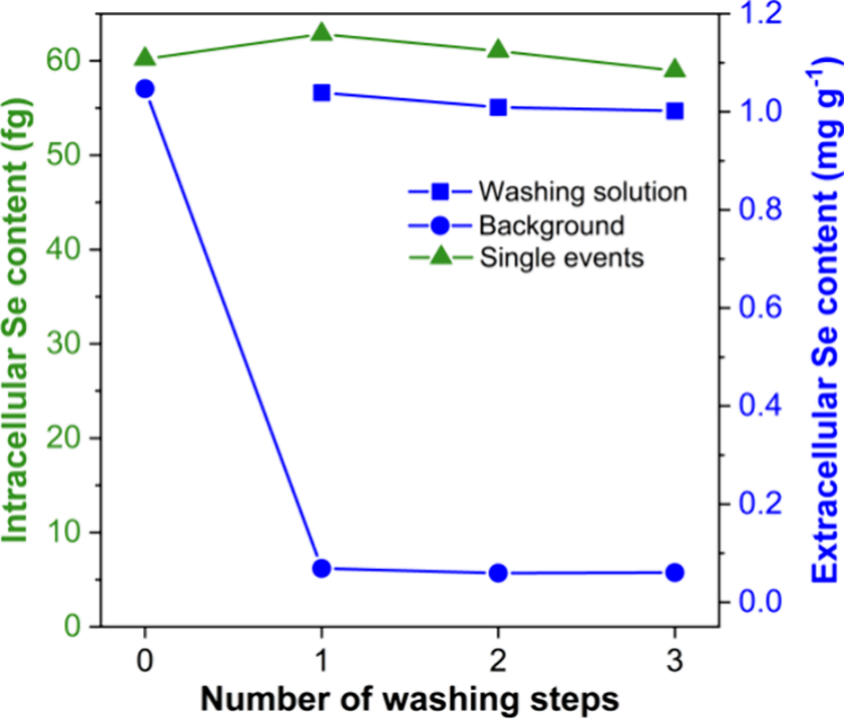
Evolution of the intra- and extracellular (both measuring the average background signal and the washing solution) Se contents depending on the number of washing steps.

After the determination of the extracellular Se contents, the accuracy of the different calibration strategies was evaluated by comparing the total Se contents (intracellular + extracellular) measured via SC-ICP-MS, the bulk ICP-MS results obtained after cell digestion (2.030 ± 0.020 mg g^–1^, n = 3), and the certified value (2.031 ± 0.070 mg g^–1^), as SELM-1 is certified for the total Se concentration. The results are summarized in Figure 5 and Table S3 of the Supporting Information.

**Figure 5:**
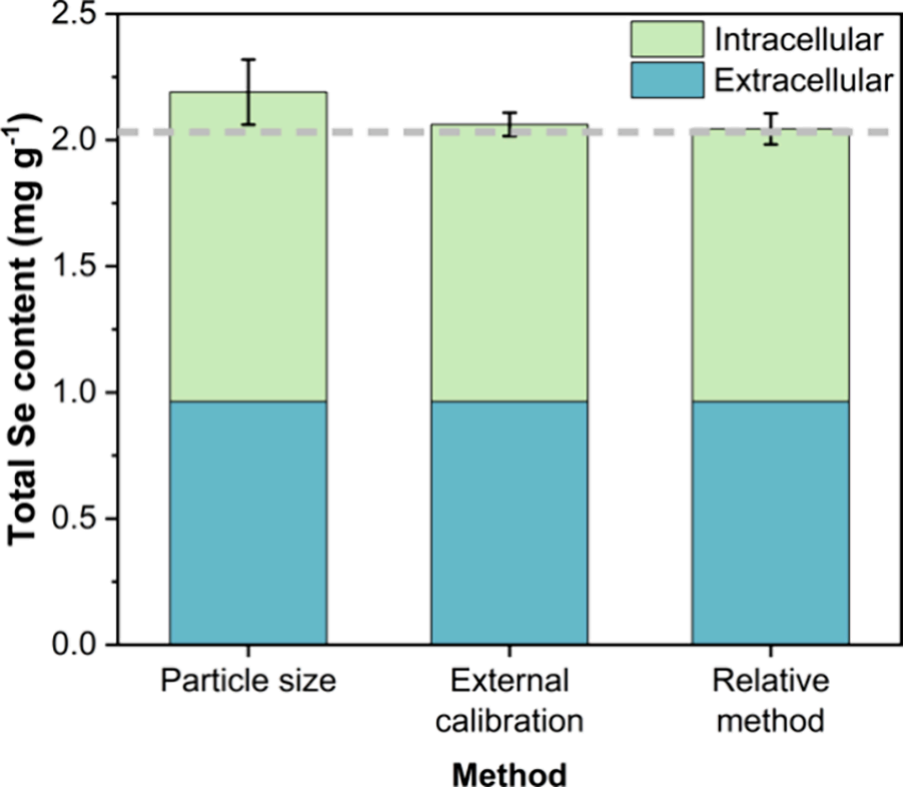
Graphical representation of the total (intra- and extracellular) Se contents. The error bars represent the standard deviation of five working sessions. The dashed line represents the reference value of the certificate (total Se content).

As can be seen, the NP-based calibration strategies for intracellular Se content determination are not only more direct and user-friendly (no measurement of ionic standards is required), but the results are more repeatable between working sessions and accurate as compared to the bulk analysis and certified value (R% of 102 ± 2% and 101 ± 3% for the external calibration and relative method, respectively). There also seems to be a clear trend for the particle size method relying on the use of AuNPs for the determination of the TE to overestimate the intracellular, and subsequently, the total Se content (R% = 108 ± 6%, ranging from 101% to 118%). This bias may not be compensated for when the results are obtained during a single measurement session, even if multiple replicates are measured. These results reinforce the need to develop alternative calibration strategies that do not rely on the use of ionic standard solutions for single-entity calibration, or at least not through prior determination of the TE. In this context, a microdroplet generator could yield accurate results, but the need for an additional device makes the approach less straightforward.^19,32^ The results obtained in this work also demonstrate the potential of directly and simultaneously determining both the intra- and extracellular contents via SC-ICP-MS.

## Conclusions

In this work, novel NP-based calibration strategies for quantitative SC-ICP-MS analysis have been explored. The fundamental principle of these approaches is to rely on entities (NPs of the target analyte, well-characterized in terms of size, density and chemical composition) for calibration, rather than on ionic standard solutions. Furthermore, accurate intra- and extracellular Se contents have been obtained directly and simultaneously for the first time. For method validation, we relied on the analysis of the SELM-1 yeast cell CRM, consisting of Se-enriched yeast, and certified for its total Se contents (intracellular + extracellular Se). By comparing the results obtained in each case to the certified Se concentration of a suspension of SELM-1, the superior performance of novel NP-based and TE-independent approaches has been demonstrated. These methodologies are expected to be applicable to other cell types, such as mammalian cells or bacteria.

As the field evolves and a greater number of NPs of different types and sizes become available, it is the authors’ view that there is an opportunity for a paradigm shift in which the determination of the ionic contents should rely on calibration using ionic standard solutions (extracellular contents), but the determination of the elemental contents in discrete entities, such as cells (intracellular contents), should ideally be based on calibration using standard entities, when available: ions with ions, entities with entities.

## Supplementary Material



## Notes

Instrument settings. Calibration curve parameters for SC-ICP-MS analysis. Intra- and extracellular Se results obtained via SC-ICP-MS analysis, total Se concentrations obtained via solution-based bulk ICP-MS analysis, and certified total Se concentration (PDF)
