# Supplementary material for: Ions with Ions, Entities with Entities: A Proof-of-Concept Study Using the SELM‑1 Yeast Certified Reference Material for Intra- and Extracellular Se Quantification via Single-Cell ICP-Mass Spectrometry
Source: Anal Chem. 2025 Jun 7;97(26):13922–9. doi: 10.1021/acs.analchem.5c01588 (PMC12242907; doi:10.1021/acs.analchem.5c01588)
Supplement: Supplementary file 1 [file ac5c01588_si_001.pdf]

# Supporting Information

## Ions with ions, entities with entities: a proof-of-concept study using the SELM-1 yeast certified reference material for intra- and extracellular Se quantification via single-cell ICP-mass spectrometry

Antonio Bazo, Eduardo Bolea-Fernandez,* Ana Rua-Ibarz, Maite Aramendía, Martín Resano*

University of Zaragoza, Department of Analytical Chemistry, Aragon Institute of Engineering Research (I3A), Zaragoza, Spain, 50009.

*Corresponding Authors
Eduardo Bolea-Fernandez, Email: ebolea@unizar.es
Martín Resano, E-mail: mresano@unizar.es

**Table of contents for Supporting Information**

Table S1. Instrument settings and data acquisition parameters for the NexION 5000 ICP-MS/MS instrument operated in single-cell mode.

Table S2. Equation and $R^2$ of the SeNPs external calibration curves obtained during the different working sessions.

Table S3. Intra- and extracellular Se results were obtained in this work via SC-ICP-MS analysis, total Se concentrations were obtained via solution-based bulk ICP-MS analysis, and certified total Se concentration (SELM-1 certificate). Uncertainties are expressed as standard deviations.

**Table S1.** Instrument settings and data acquisition parameters for the NexION 5000 ICP-MS/MS instrument operated in single-cell mode.

| Measurement conditions | |
|---|---|
| RF Power, W | 1600 |
| Nebulizer gas flow, L $min^{-1}$ | 0.3 |
| Make up gas flow, L $min^{-1}$ | 0.9 |
| Plasma gas flow, L $min^{-1}$ | 16 |
| Auxiliary gas flow, L $min^{-1}$ | 1.2 |
| Detection mode | Q3 Only |
| Nuclide monitored | $^{82}Se$ |
| Dwell time, µs | 100 |
| Acquisition time, s | 100 |
| RPq | 0.25 |
| **Instrumental setup** | |
| Spray chamber | Asperon™ |
| Nebulizer | Single-Cell MicroFlow |
| Introduction system | Syringe pump |
| Uptake rate, mL $min^{-1}$ | 0.02 |
| **NP parameters for calculations** | |
| AuNP density, g $cm^{-3}$ | 19.32 |
| SeNP density, g $cm^{-3}$ | 4.79 |

**Table S2.** Equation and $R^2$ of the SeNPs external calibration curves obtained during the different working sessions.

| Session | Equation | $R^2$ |
|---|---|---|
| 1 | $y = 26.18x - 6.15$ | 0.9997 |
| 2 | $y = 27.42x + 2.11$ | 1.0000 |
| 3 | $y = 26.36x - 5.53$ | 0.9998 |
| 4 | $y = 27.46x - 3.26$ | 0.9999 |
| 5 | $y = 26.27x + 0.23$ | 1.0000 |

**Table S3**. Intra- and extracellular Se results were obtained in this work *via* SC-ICP-MS analysis, total Se concentrations were obtained *via* solution-based bulk ICP-MS analysis, and certified total Se concentration (SELM-1 certificate). Uncertainties are expressed as standard deviations.

| Intracellular Se content (fg cell$^{-1}$) | | | | | | |
|---|---|---|---|---|---|---|
| **Method** | **Session 1** | **Session 2** | **Session 3** | **Session 4** | **Session 5** | **Average** |
| Particle size | 60.8 ± 23.3 | 79.3 ± 30.9 | 65.3 ± 23.7 | 72.9 ± 33.3 | 66.1 ± 31.1 | 68.87 ± 7.26 |
| External calibration | 61.7 ± 23.6 | 61.6 ± 24.0 | 62.7 ± 22.7 | 59.9 ± 31.4 | 62.4 ± 25.5 | 61.67 ± 1.07 |
| Relative method | 56.4 ± 21.6 | 60.7 ± 23.7 | 61.0 ± 22.3 | 60.4 ± 32.7 | 64.9 ± 25.7 | 60.68 ± 3.01 |
| **Intracellular Se content (mg g$^{-1}$)** | | | | | | |
| **Method** | **Session 1** | **Session 2** | **Session 3** | **Session 4** | **Session 5** | **Average** |
| Particle size | 1.08 ± 0.41 | 1.41 ± 0.55 | 1.16 ± 0.42 | 1.30 ± 0.59 | 1.18 ± 0.55 | 1.23 ± 0.13 |
| External calibration | 1.10 ± 0.42 | 1.10 ± 0.43 | 1.12 ± 0.40 | 1.07 ± 0.56 | 1.11 ± 0.45 | 1.10 ± 0.02 |
| Relative method | 1.00 ± 0.38 | 1.08 ± 0.42 | 1.09 ± 0.40 | 1.08 ± 0.58 | 1.16 ± 0.46 | 1.08 ± 0.05 |
| **Extracellular Se content (mg g$^{-1}$)** | | | | | | |
| **Method** | **Session 1** | **Session 2** | **Session 3** | **Session 4** | **Session 5** | **Average** |
| Direct determination | 0.97 ± 0.32 | 0.99 ± 0.28 | 0.98 ± 0.30 | 0.91 ± 0.24 | 0.97 ± 0.27 | 0.96 ± 0.03 |
| **Total Se content (mg g$^{-1}$)** | | | | | | |
| **Method** | **Session 1** | **Session 2** | **Session 3** | **Session 4** | **Session 5** | **Average** |
| Particle size | 2.05 ± 0.44 | 2.40 ± 0.57 | 2.14 ± 0.45 | 2.21 ± 0.60 | 2.15 ± 0.57 | 2.19 ± 0.13 |
| External calibration | 2.07 ± 0.45 | 2.09 ± 0.45 | 2.10 ± 0.43 | 1.98 ± 0.57 | 2.08 ± 0.47 | 2.06 ± 0.47 |
| Relative method | 1.97 ± 0.42 | 2.07 ± 0.44 | 2.07 ± 0.42 | 1.99 ± 0.59 | 2.13 ± 0.48 | 2.04 ± 0.48 |
| **Recovery (%)** | | | | | | |
| **Method** | **Session 1** | **Session 2** | **Session 3** | **Session 4** | **Session 5** | **Average** |
| Particle size | 101 ± 22 | 118 ± 28 | 106 ± 22 | 109 ± 30 | 106 ± 28 | 108 ± 6 |
| External calibration | 102 ± 22 | 103 ± 22 | 103 ± 21 | 97 ± 28 | 103 ± 23 | 102 ± 2 |
| Relative method | 97 ± 21 | 102 ± 22 | 102 ± 21 | 98 ± 29 | 105 ± 24 | 101 ± 3 |

| Bulk analysis (mg g$^{-1}$) | | | | |
|---|---|---|---|---|
| **Method** | **Session 1** | **Session 2** | **Session 3** | **Average** |
| Direct bulk determination | 2.05 ± 0.43 | 2.01 ± 0.47 | 2.04 ± 0.45 | 2.03 ± 0.02 |
| **Certified value (mg g$^{-1}$):** 2.031 ± 0.070 | | | | |
